# Batch and Continuous Flow Preparation of Hantzsch 1,4-Dihydropyridines under Microwave Heating and Simultaneous Real-time Monitoring by Raman Spectroscopy. An Exploratory Study

**DOI:** 10.3390/molecules19079986

**Published:** 2014-07-09

**Authors:** Sylvain Christiaens, Xavier Vantyghem, Marilena Radoiu, Jean Jacques Vanden Eynde

**Affiliations:** 1 Laboratory of Organic Chemistry, University of Mons-UMONS, 23 Place du Parc, B-7000 Mons, Belgium; E-Mails: spit06@hotmail.com (S.C.); mxiano@hotmail.com (X.V.); 2 SAIREM SAS, 12 porte du Grand Lyon, F-01702 Neyron, France; E-Mail: mradoiu@sairem.com

**Keywords:** 1,4-DHPs, flow chemistry, Hantzsch pyridines, microwave, Raman spectroscopy, real-time monitoring

## Abstract

Dialkyl 1,4-dihydro-2,6-dimethylpyridine-3,5-dicarboxylates have been prepared in a batch mode under conventional heating as well as under continuous flow conditions in the Miniflow 200SS, Sairem’s microwave-assisted batch and continuous flow equipment. Real-time monitoring of the reactions by Raman spectroscopy enabled to compare both heating modes and to determine (optimized) reaction times.

## 1. Introduction

Many organic reactions are now routinely run in domestic or dedicated microwave ovens and the subject is regularly reviewed [1,2,3,4,5,6]. High yields, high purity, high selectivity, and shorter reaction times are often claimed to be associated with microwave-assisted syntheses. However, the experiments are generally only conducted on a few milligrams of chemicals because of the penetration depth of the microwaves. The efficiency of the heating is also highly dependent on the nature of the reaction mixture (solvents and reagents). Due to those restrictions, scaling-up emerges as a challenge that is difficult to address in the batch mode. Hamelin *et al.* [7] and Loupy *et al.* [8] used a one-liter quartz reactor in a Prolabo oven [9] to prepare around 200 g of dioxalanes, dithiolanes, or oxathiolanes and octyl acetate respectively. Leadbeater *et al.* [10] synthesized one mole (196 g) of 4-acetylbiphenyl by employing a 3-liter round-bottom flask in a CEM Mars oven.

**Figure 1:**
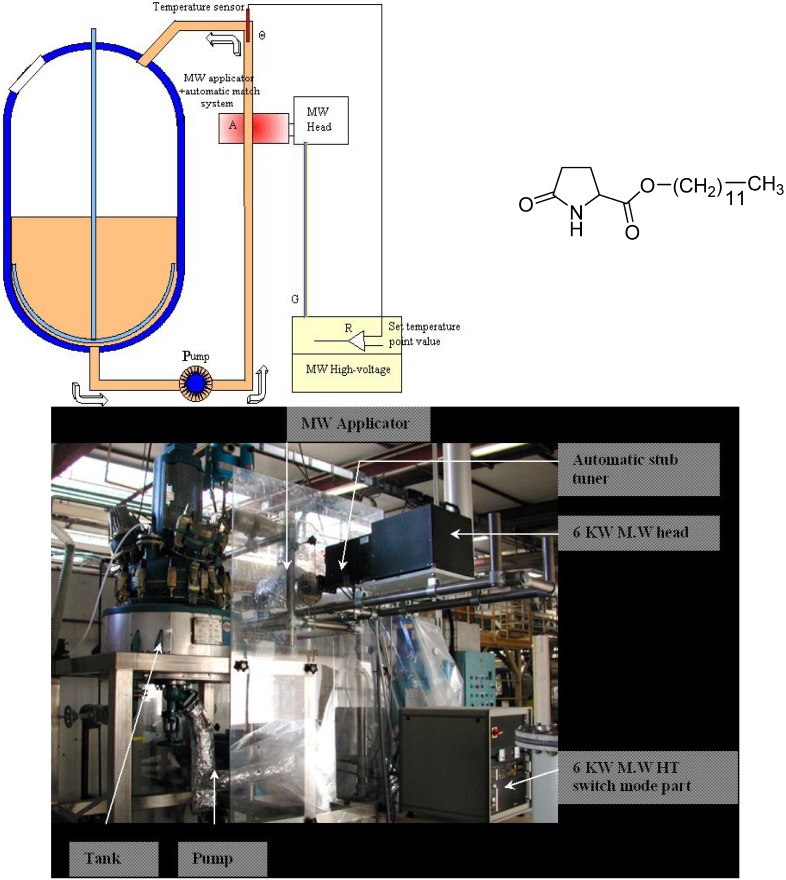
Sairem microwave-assisted reactor for Laurydone^®^ production (courtesy of Sairem SAS).

More interestingly, another way to scale up microwave-assisted reactions is to shift to continuous flow conditions in open or closed loop modes [11,12]. The idea was initially developed by Strauss *et al.* [13] at the end of the 1980s and led to real industrial applications among which the microwave-assisted synthesis of Laurydone^®^, a moisturizer found in lipsticks and other make-up products (see Figure 1).

In such continuous flow systems, the reaction mixture is transferred from a reservoir and transits in the microwave cavity before ending in a collector. Thus appropriate adjustment of the flow rates is a requisite to ensure that the reagents are completely consumed before they are pumped out of the reactor. To facilitate this, kinetic data for the concerned reactions under closely related experimental conditions must be collected. This is far from a trivial situation especially if sampling during microwave-assisted reactions requires the irradiation to be stopped and the reactor cooled for safety reasons. To the best of our knowledge there are two spectroscopic methods that allow acquisition of analytical data in real-time for syntheses performed in microwave equipment:

- Infrared spectroscopy: in 1999 Te-As-Se based optical glass fibers were developed at the University of Rennes 1, France by Lucas *et al.* [14] and used as evanescent wave chemical sensors by Perio [15] in the Hamelin group. The fibers were directly introduced in the reaction media and infrared spectra were periodically recorded. In particular the disappearance of the carbonyl band due to the C=O vibration in 3-pentanone or cyclohexanone was monitored when those ketones were reacted with trimethyl orthoformate in the presence of Montmorillonite K10 clay or with mercaptoethanol in the presence of *para*-toluenesulfonic acid. Some patents [16,17,18] cover the subject, but for unknown reasons the technology has fallen into oblivion.
- Raman spectroscopy: the method does not require an immersion of the probe into the reactor but only a contact to the exterior wall. A first report [19] on the subject was published in 1995 and a full description of the instrumentation and its setup has been disclosed by Pivonka and Empfield [20] in 2004. These publications have been followed by the work of Leadbeater *et al.* [21,22,23,24,25,26,27], who used a CEM oven modified to allow introduction of a Raman probe into the microwave cavity.

In this work we report the results obtained in the preparation of Hantzsch 1,4-dihydropyridines in a prototype microwave equipment designed and manufactured by Sairem SAS, France. The initial reactions were performed in batch mode and monitored in real-time by Raman spectroscopy. The data obtained have been further exploited to extrapolate experimental conditions for continuous flow processing under microwave irradiation.

## 2. Results and Discussion

### 2.1. Choice of Reaction

**Scheme 1:**
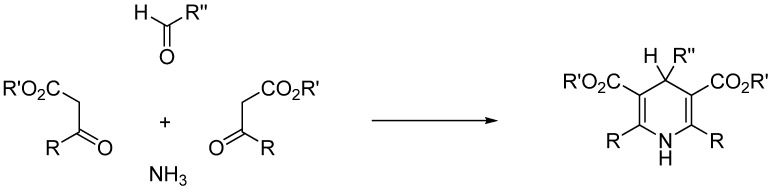
Preparation of Hantzsch 1,4-dihydropyridines.

Dialkyl 1,4-dihydropyridine-3,5-dicarboxylates (1,4-DHPs) are known as Hantzsch 1,4-dihydropyridines. They are generally obtained by the Hantzsch pyridine synthesis [28] from an aldehyde, a β-ketoester (2 equivalents), and a source of ammonia (Scheme 1). The reaction has been reviewed [29] and mention should be made that the Hantzsch pyridine synthesis has already been scaled up under flow conditions and microwave irradiation [30,31].

Diethyl 1,4-dihydro-2,6-dimethylpyridine-3,5-dicarboxylate (**1**: R = CH_3_, R' = C_2_H_5_, R'' = H) is one of the simplest representatives of the family. Due to its structural similarity to coenzyme nicotinamide adenine dinucleotide (NADH) it is often used [32] as a mimic of the enzyme in biological studies. Several 1,4-dihydropyridines (1,4-DHPs) are used as calcium channel modulators for the treatment of hypertension and angina pectoris [33]. Other derivatives have been reported to exhibit antitrypanosomal, antitubercular, anticancer, antiplasmodial, antileishmanial, antibacterial, and antioxidant activities [33].

### 2.2. Materials and Methods

#### 2.2.1. The Raman Spectrometer

**Figure 2:**
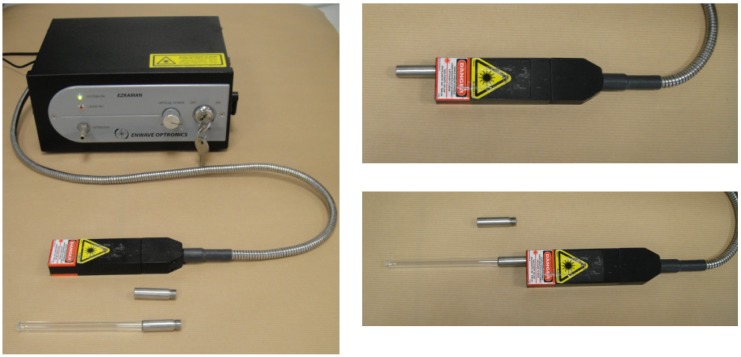
The Enwave Optronics EZRaman^TM^-M spectrometer.

The Raman spectra were recorded using an Enwave Optronics EZRaman^TM^-M spectrometer, (Figure 2). The spectrometer is equipped with a 785 nm frequency stabilized excitation laser source tunable from 0 to 300–400 mW and a linear CCD array covering a range of wavelength from 250 to 2350 cm^−1^. The fiber-optic probe is composed of a 100 μm excitation fiber and a 200 µm collection fiber. The contact measuring stainless lens-tube can be readily replaced by a quartz tube.

#### 2.2.2. The Conventional Heating System

**Figure 3:**
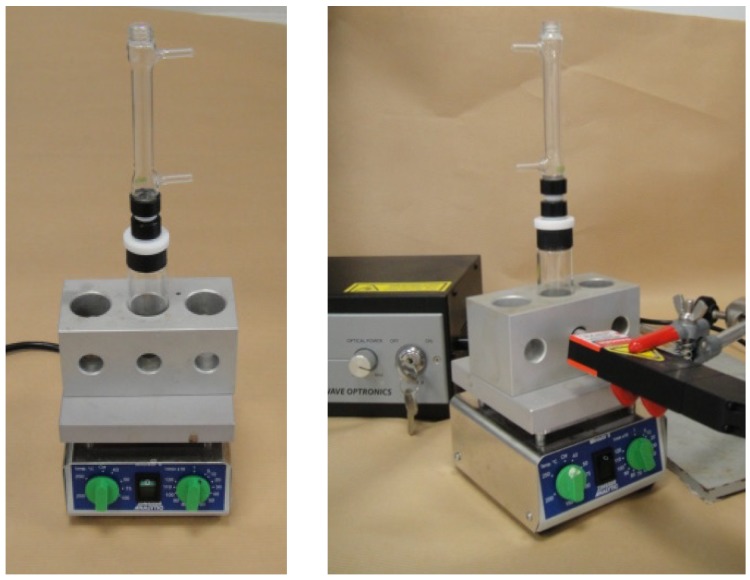
The Zinsser-Minilab^®^ system.

In addition to classical glassware equipment, we also performed reactions by using a Zinsser-Minilab^®^ system, Figure 3. Holes in the heating block allowed introduction of the lens-tube of the Raman spectrometer in direct contact with the reactor.

#### 2.2.3. The Microwave (Dielectric) Heating Method & Equipment

**Figure 4:**
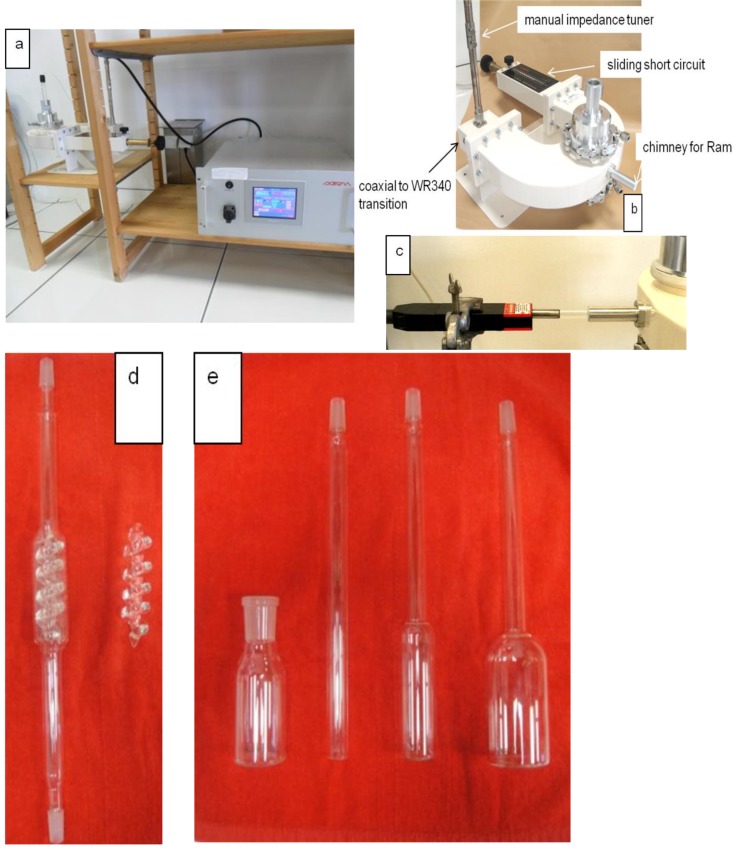
The Sairem prototype microwave equipment. (**a**) The MiniFlow 200 SS consists of a microwave solid state (transistor) generator, programmable logic controller, and fiber optic thermometer; microwave parameters (forward power, reflected power), time, and temperature are controlled via a touch screen digital display. (**b**) The U-shaped WR340 waveguide consists of a manual coaxial impedance tuner, a coaxial to WR340 transition, a monomode (transversal electric) cavity (continuous flow or batch reactors are placed within the cavity) with a lateral chimney for Raman spectrometer probe, and manual sliding short circuit for reflected power optimization. (**c**) Detail of lateral chimney and Raman spectrometer probe. (**d**) Custom made continuous flow reactor (internal volume of 40 mL) and detail of the mixing chamber, borosilicate glass. (**e**) Different custom made batch reactors, borosilicate glass.

The experiments were performed in a prototype 2.45 GHz microwave equipment, MiniFlow 200SS (Figure 4) designed and manufactured by Sairem SAS, Neyron, France.

The device is characterized by the following features:

- 2.45 GHz microwaves are delivered in continuous wave (CW) by a solid state (transistor) microwave generator; the microwave power can be adjusted from 1 W to 200 W in steps of 1 W;
- The microwave power is transmitted from the solid state generator to the U-shaped waveguide using a coaxial cable 1 m long;
- The device enables measurement (via a built-in circulator) and adjustment (using the manual sliding short circuit and if necessary the manual impedance tuner) of the reflected microwave power so that maximum energy is available and transferred into the reaction medium with limited loss;
- The temperature inside the reactors was measured directly in the mixture with a fiber optic temperature sensor (Neoptix, Québec City, Québec, Canada) and after the heating ramp period, the power of the microwave irradiation was automatically adjusted in order to maintain the chosen final reaction temperature for a given period;
- A lateral chimney allows the introduction of a probe into the cavity of the U-shaped waveguide and more specifically the introduction of a quartz Raman probe;
- The same equipment can be used to perform reactions under both batch and continuous flow.

The performances of the Sairem prototype equipment have been compared with those of the Biotage Initiator^®^ oven.

### 2.3. Preparation of a Reference Sample 1 under Conventional Heating

To obtain a reference sample of diethyl 1,4-dihydro-2,6-dimethylpyridine-3,5-dicarboxylate (**1**) we reproduced the protocol reported by Norcross *et al.* [34]. A mixture of aqueous formaldehyde (37% in water and 10%–15% of methanol; 1.4 mL; 18.70 mmol), ethyl acetoacetate (5.0 mL; 39.06 mmol), and concentrated aqueous ammonia (25% in water; 6.2 mL; 82.97 mmol) in ethanol (2.0 mL) was heated at reflux for 30 min. After cooling the precipitate was filtered, rinsed with well-chilled ethanol (0–5 °C; 5–10 mL) and recrystallized from ethanol. The procedure enabled isolation of the expected 1,4-DHP but was not appropriate for monitoring by Raman spectroscopy. Indeed after the addition of the aqueous solution of ammonia, the reaction mixture became biphasic and the 1,4-DHP precipitated during the heating period. Therefore we preferred to use a methanolic solution of ammonia, to replace ethanol by methanol, and to increase the volume of solvent. The protocol adopted in this work is thus described hereafter. A mixture of aqueous formaldehyde (37% in water and 10%–15% of methanol; 0.7 mL; 9.35 mmol), ethyl acetoacetate (2.5 mL; 19.53 mmol), and a methanolic solution of ammonia (15.5% in methanol; 3.0 mL; 21.02 mmol) in methanol (4.0 mL) was heated at 50 °C for the appropriate period of time (see text). Under such conditions (solvents and temperature) the reaction medium was homogeneous and the final DHP did not precipitate.

### 2.4. Preliminary Study by Raman Spectroscopy

We first recorded the Raman spectra of the solvents, reagents, and the final 1,4-DHP **1**. From the spectra it clearly appeared that the disappearance of the β-ketoester could be visualized by monitoring the decrease of the carbonyl band at 1735 cm^−1^. The appearance of the DHP could be monitored by observing the increase of the corresponding carbonyl band at 1644 cm^−1^. There is no interference between those two peaks and other absorption bands.

In order to collect quantitative data, we recorded the intensity of those two peaks as a function of the concentration in a mixture of water and methanol (corresponding to the experimental conditions of the preparation of the DHP). Because the intensities of the peaks for a given concentration were not always perfectly reproducible, we found it more accurate to correlate the concentrations to the ratio of the intensity of the carbonyl peaks to the intensity of a peak of methanol at 1026 cm^−1^. This way linear relationship could be readily established. Spectra were recorded on samples heated at 50 °C (*vide supra*).

### 2.5. Reactions in Batch Mode

#### 2.5.1. Reaction in a Mixture of Methanol and Water

**Figure 5:**
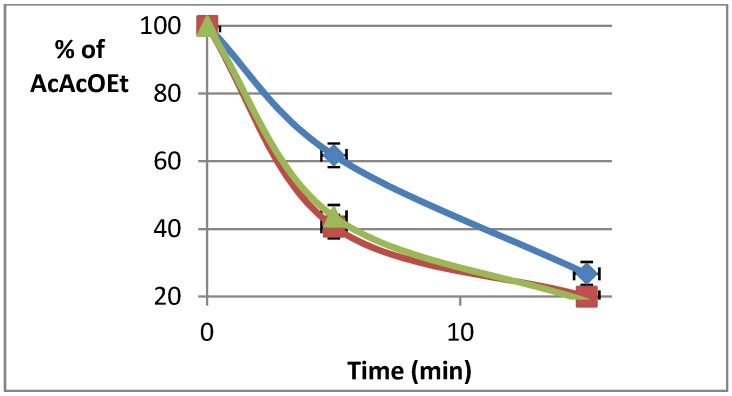
Disappearance of ethyl acetoacetate as a function of time for reactions performed at 50 °C under conventional heating (**blue**), and dielectric (microwave) heating: Sairem Miniflow 200SS (**green**), Biotage Initiator^®^ (**red**).

The reaction between aqueous formaldehyde, ethyl acetoacetate, and ammonia in methanol was performed under conventional heating (Zinsser-Minilab^®^ system) or dielectric heating in both sets of microwave equipment. The disappearance of the β-ketoester was monitored (Figure 5) in real-time by Raman spectroscopy for the synthesis under conventional heating and in the MiniFlow 200SS. In the case of the synthesis carried out in the Biotage oven, the reactor was taken out of the irradiation cavity and the spectrum was immediately recorded.

For reasons of clarity, only data corresponding to the composition of the medium after 0, 5, and 15 min are presented. Figure 5 clearly shows that dielectric (microwave) heating favors the disappearance of the β-ketoester. This is however related to the period of time required to attain the reaction temperature fixed at 50 °C. Under conventional heating the final temperature was reached after a period of about 150 s whereas that period was reduced to 12 s using the Miniflow 200 SS and 24 s using the Biotage Initiator^®^.

#### 2.5.2. Reaction in a Mixture of Acetonitrile and Water

It is well known that solvents do not absorb electromagnetic waves in the same way. This concept is expressed by the loss tangent factor (tan δ) [35]. In our hands, with the MiniFlow 200SS, for a forward power of 100 W, the measured reflected power (after optimization with the manual sliding short circuit and the manual impedance tuner) was 4 W for methanol and 31 W for acetonitrile (glass reactor with an outer diameter of 30 mm and filled with 25 mL of solvent). Accordingly, reactions in which the β-ketoester was dissolved in acetonitrile were slower than those involving the β-ketoester dissolved in methanol. However, this result cannot be directly linked to a “microwave effect” because we observed that the same situation occurred for syntheses performed under conventional heating.

#### 2.5.3. Extension to the Preparation of Other 1,4-DHPs

Experimentally we observed that *tert*-butyl acetoacetate was consumed slower than the ethyl and methyl analogs (methanol was used as a solvent). This was true under conventional heating and dielectric heating; steric effects can be reasonably invoked to explain this observation.

### 2.6. Reaction under Continuous Flow Conditions

**Figure 6:**
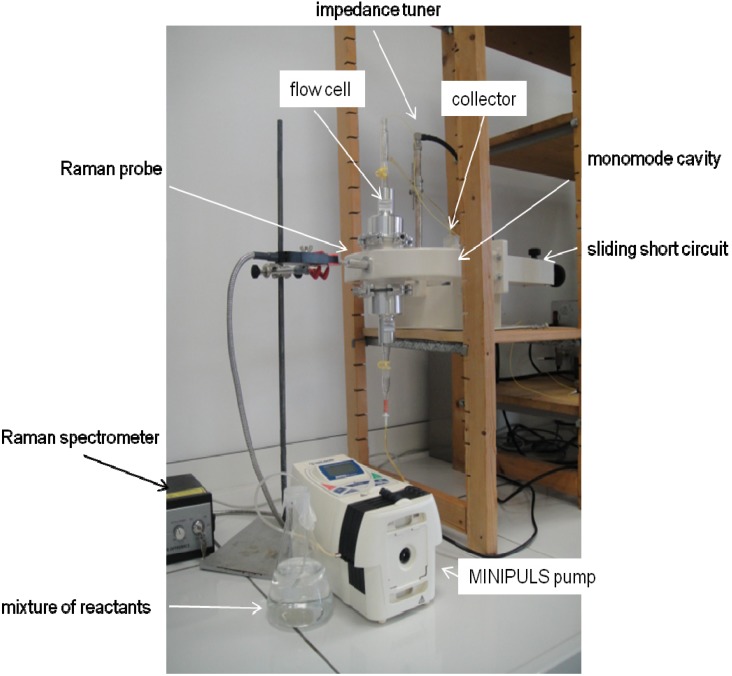
Setup of a continuous flow synthesis under microwave irradiation in the MiniFlow 200SS.

**Figure 7:**
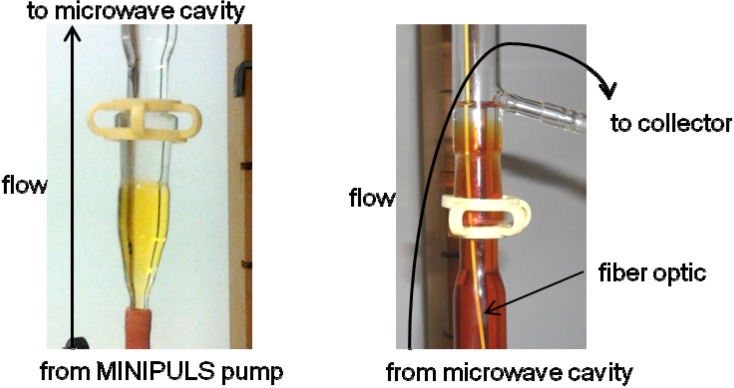
Connecting vessels before (**left**) and after (**right**) the microwave cavity.

When working in batch mode we found that more than 80% of the starting β-ketoester was consumed within 15 min under microwave irradiation at 50 °C. The volume of the reaction chamber of the flow cell was 40 mL. Therefore we decided to irradiate the reaction medium for approximately 30 min by adjusting the flow rate to 1.4 mL/min (MINIPULS evolution^®^ peristaltic pump, Gilson, Middleton, WI, USA). To start the experiment (Figure 6), we filled the connecting tubes, connecting vessels, and reaction chamber with methanol. The flow was then started pumping methanol whilst maintaining a temperature of 50 °C in the chamber. The temperature was obtained within 4 min. We then started pumping the reaction medium and interestingly, due to the slow flow rate, we were able to follow visually the evolution of the reaction (Figure 7). As the reaction mixture progressed through the flow cell, the clear translucent solution of methanol at the bottom of the flow cell was replaced by a yellow reaction mixture and a reddish colour developed at the top exit of the flow cell. More importantly, Raman spectra were recorded at the bottom (before microwaves) and at the top of the cell (after microwaves) but also in the middle (inside the microwave field). Those spectra were sufficiently resolved to indicate the disappearance of the ketoester after the passage in the microwave cavity. The pumping was conducted during 3 h period after which methanol was pumped to flush the reaction medium out of the flow cell. The microwave irradiation was stopped afterwards. At the end we isolated 50 g of 1,4-DHP which represents a yield of approximately 80%.

## 3. Experimental Section

### 3.1. General

Reactions under conventional heating were conducted in round-bottom flasks or in the Zinsser-Minilab^®^ system (Zinsser Analytic, Frankfurt, Germany). Reactions under microwave heating were conducted in the Sairem MiniFlow 200SS oven (Sairem, Neyron, France) or in the Biotage Initiator^®^ oven (Biotage, Uppsala, Sweden). Reactions under flow conditions were performed using a MINIPULS evolution^®^ peristaltic pump (Gilson, Middleton, WI, USA).

^1^H-NMR spectra were obtained using a Bruker AMX instrument (300 MHz) and TMS as internal reference. IR spectra were recorded on a Perkin-Elmer FTIR 1760K and Raman spectra were recorded using the Enwave Optronics EZRaman^TM^-M spectrometer (Enwave Optronics Inc, Irvine, CA, USA). Solvents and reagents were commercially available from Sigma-Aldrich (Diegem, Belgium) or Acros Organics (Geel, Belgium) and were used without further purification. All compounds have been described in the literature [29] and their structure was confirmed by comparison with data recorded on samples available in our laboratory [36].

### 3.2. Chemistry

The following general procedure was used to perform reactions in the batch mode: a mixture of aqueous formaldehyde (37% in water and 10%–15% of methanol; 0.7 mL; 9.35 mmol), a β-ketoester (19.53 mmol), and a methanolic solution of ammonia (15.5% in methanol; 3.0 mL; 21.02 mmol) in methanol (4.0 mL) was heated at 50 °C for the appropriate period of time (see text). After cooling the precipitate was filtered, rinsed with well-chilled ethanol (0–5 °C; 5–10 mL) and recrystallized from ethanol. Acetonitrile was used as the sovent (instead of methanol) in one experiment (see Section 2.5.2). Yields: 75%–90%.

For the reaction performed under flow conditions, a cold (5 °C) stock solution of a mixture of aqueous formaldehyde (37% in water and 10%–15% of methanol; 17.5 mL; 233.75 mmol), ethyl acetoacetate (62.5 mL; 488.25 mmol), and a methanolic solution of ammonia (15.5% in methanol; 75.0 mL; 525.5 mmol) in methanol (100.0 mL) was prepared in an Erlenmeyer. The solution was pumped at a flow rate adjusted to 1.4 mL/min and collected after irradiation in another Erlenmeyer. After cooling the precipitate was filtered, rinsed with well-chilled ethanol (0–5 °C; 5–10 mL) and recrystallized from ethanol. Yield: 80%.

## 4. Conclusions

During this exploratory work we have demonstrated that Raman spectroscopy can readily and advantageously be used to monitor in real-time organic reactions under conventional and dielectric heating in a batch mode. Homogeneous clear mixtures are however required to ensure a good quality of the spectra.

The MiniFlow 200SS microwave equipment enabled reactions to be performed in batch mode as well as under continuous flow. We observed that experimental conditions established for the batch mode experiment could be directly extrapolated to continuous flow conditions. In this latter case, usinga slow flow rate, spectra could be recorded in real-time.

## References

[1] Leadbeater N.E., McGowan C.B. (2013). Laboratory Experiments Using Microwave Heating.

[2] Hassan H.M.A., Harakeh S., Sakkaf K.A., Denetiu I. (2012). Progress in microwave-aided chemical synthesis. Aust. J. Chem..

[3] Kruithof A., Ruijter E., Orru R.V.A. (2011). Microwave-assisted multicomponent synthesis of heterocycles. Curr. Org. Chem..

[4] Appukkuttan P., Mehta V.P., van Der Eycken E.V. (2010). Microwave-assisted cycloaddition reactions. Chem. Soc. Rev..

[5] Strauss C.R. (2009). A strategic, “green” approach to organic chemistry with microwave assistance and predictive yield optimization as core, enabling technologies. Aust. J. Chem..

[6] Bogdal D., Loupy A. (2008). Application of microwave irradiation to phase-transfer catalyzed reactions. Org. Process Res. Dev..

[7] Perio B., Dozias M.-J., Hamelin J. (1998). Ecofriendly fast batch synthesis of dioxolanes, dithiolanes, and oxathiolanes without solvent under microwave irradiation. Org. Process Res. Dev..

[8] Cleophax J., Liagre M., Loupy A., Petit A. (2000). Application of focused microwaves to the scale-up of solvent-free organic reactions. Org. Process Res. Dev..

[B9-molecules-19-09986] 9.The Prolabo mark, popular in France in the 1990s, has now disappeared.

[10] Leadbeater N.E., Williams V.A., Barnard T.M., Collins M.J. (2006). Open-vessel microwave-promoted Suzuki reactions using low levels of palladium catalyst: Optimization and scale-up. Org. Process Res. Dev..

[11] Baxendale I.R., Hayward J.J., Ley S.V. (2007). Microwave reactions under continuous flow conditions. Comb. Chem. High T. Scr..

[12] Barbry D., Vanden Eynde J.J., Bazureau J.P., Draye M. (2011). Continuous flow organic synthesis under microwave heating. Ultrasound and Microwaves: Recent Advances in Organic Chemistry.

[13] Strauss C.R., Trainor R.W. (1995). Developments in microwave-assisted organic chemistry. Aust. J. Chem..

[14] Hocde S., Boussard-Pledel C., le Coq D., Fonteneau G., Lucas J., Saad M., Harrington J.A. Remote analysis using IR glass fibers. Infrared Optical Fibers and Their Applications, Proceedings of the Meeting of the Society of Photo-optical Instrumentation Engineers.

[15] Perio B. (1999). Solvent-Free Protection of Carbonyl Groups under Microwave Irradiation: A Clean and Competitive Process. Ph.D. thesis.

[16] Brooks H.J., Mortenson M.G., Blum B.J. (2005). Controlling Chemical Reactions by Spectral Chemistry and Spectral Conditioning. U.S. Patent.

[17] McManus M.E., Collins M.J., Collins M.J. (2006). Spectroscopy-Base Real-Time Control for Microwave-Assisted Chemistry. U.S. Patent.

[18] King E.E. (2007). Real-Time Imaging and Spectroscopy during Microwave Assisted Chemistry. U.S. Patent.

[19] Stellman C.M., Aust J.F., Myrick M.L. (1995). *In situ* spectroscopic study of microwave polymerization. Appl. Spectrosc..

[20] Pivonka D.E., Empfield J.R. (2004). Real-time *in situ* Raman analysis of microwave-assisted organic reactions. Appl. Spectrosc..

[21] Barnard T.M., Leadbeater N.E. (2006). Real-time monitoring of microwave-promoted organometallic ligand-substitution reactions using *in situ* Raman spectroscopy. Chem. Commun..

[22] Leadbeater N.E., Smith R.J. (2006). Real-time monitoring of microwave-promoted Suzuki coupling reaction using *in situ* Raman spectroscopy. Org. Lett..

[23] Leadbeater N.E., Smith R.J., Barnard T.M. (2007). Using *in situ* Raman monitoring as a tool for rapid optimization and scale-up of microwave-promoted organic synthesis: Esterification as an example. Org. Biomol. Chem..

[24] Leadbeater N.E., Schmink J.R. (2008). Use of Raman spectroscopy as a tool for *in situ* monitoring of microwave-promoted reactions. Nat. Protoc..

[25] Schmink J.R., Holcomb J.L., Leadbeater N.E. (2008). Use of Raman spectroscopy as an *in situ* tool to obtain kinetic data for organic transformations. Chem. Eur. J..

[26] Schmink J.R., Holcomb J.L., Leadbeater N.E. (2009). Testing the validity of microwave-interfaced, *in situ* Raman spectroscopy as a tool for kinetic studies. Org. Lett..

[27] Schmink J.R., Leadbeater N.E. (2009). Probing “microwave effects” using Raman spectroscopy. Org. Biomol. Chem..

[28] Hantzsch A. (1882). Ueber die synthese pyridinartiger verbindungen aus acetessigäther und aldehydammoniak. Justus Liebigs Ann. Chem..

[29] Vanden Eynde J.J., Mayence A., Müller T.J.J. (2014). 1,3-Dicarbonyl compound as third component (Hantzsch pyridine synthesis). Science of Synthesis: Multicomponent Reactions 1.

[30] Bagley M.C., Jenkins R.L., Caterina Lubiou M., Mason C., Wood R. (2005). A simple continuous flow microwave reactor. J. Org. Chem..

[31] Baxendale I.R., Hornung C., Ley S.V., Molina J.M.M., Wikström A. (2013). Flow microwave technology and microreactors in synthesis. Aust. J. Chem..

[32] Huang Y. (2007). Hantzsch 1,4-dihydropyridine—An effective and convenient reducing agent. Synlett.

[33] Edraki N., Mehdipour A.R., Khoshneviszadeh M., Miri R. (2009). Dihydropyridines: Evaluation of their current and future pharmacological applications. Drug Discov. Today.

[34] Norcross B.E., Clement G., Weinstein M. (1969). The Hantzsch pyridine synthesis. A factorial design experiment for the introductory organic laboratory. J. Chem. Educ..

[35] Lidström P., Tierney J., Wathey B., Westman J. (2001). Microwave assisted organic synthesis—A Review. Tetrahedron.

[36] Vanden Eynde J.J., Delfosse F., Mayence A., van Haverbeke Y. (1995). Old reagents, new results: Aromatization of Hantzsch 1,4-dihydropyridines with manganese dioxide and 2,3-dichloro-5,6-dicyano-1,4-benzoquinone. Tetrahedron.

